# Assessing the Impact of Housing Features and Environmental Factors on Home Indoor Radon Concentration Levels on the Navajo Nation

**DOI:** 10.3390/ijerph17082813

**Published:** 2020-04-19

**Authors:** Sheldwin A. Yazzie, Scott Davis, Noah Seixas, Michael G. Yost

**Affiliations:** 1 Albuquerque Area Southwest Tribal Epidemiology Center, Albuquerque Area Indian Health Board, Inc., 7001 Prospect Place NE, Albuquerque, NM 87110, USA; 2 Department of Epidemiology, University of Washington, Box 357236, 1959 NE Pacific Street, Health Sciences Building, F-250D, Seattle, WA 98195, USA; scottd@uw.edu; 3 Department of Environmental & Occupational Health Sciences, University of Washington, Box 354695, 4225 Roosevelt Way NE, Seattle, WA 98195, USA; nseixas@uw.edu (N.S.); airion@u.washington.edu (M.G.Y.)

**Keywords:** Navajo Nation, Community Uranium Exposure Journey to Healing Program, radon exposure, indoor air quality, home assessment, Tribal Healthy Homes, radon measurements

## Abstract

Uranium is naturally found in the environment as a radioactive metal element with high concentrations in the Southwestern US. In this region is the Navajo Nation, which spans approximately 69,930 square kilometers. A decay product of uranium is radon gas, a lung carcinogen that has no color, odor, or taste. Radon gas may pass from soil into homes; and, indoor accumulation has been associated with geographical location, seasonality, home construction materials, and home ventilation. A home and indoor radon survey was conducted from November 2014 through May 2015, with volunteers who reported residence on the Navajo Nation. Home geolocation, structural characteristics, temperature (°C) during radon testing, and elevation (meters) were recorded. Short-term indoor radon kits were used to measure indoor radon levels. 51 homes were measured for indoor radon levels, with an arithmetic mean concentration of 60.5 Becquerels per cubic meter (Bq/m^3^) (SD = 42.7). The mean indoor radon concentrations (Bq/m^3^) by house type were: mobile, 29.0 (SD = 22.9); wood, 58.6 (SD = 36.0); hogan, 74.0 (SD = 0.0); homes constructed of cement and wood, 82.6 (SD = 3.5); and homes constructed of concrete and cement, 105.7 (SD = 55.8). A key observation is that house construction type appears to be associated with the mean home indoor radon concentration. This observation has been published in that the basic structural make-up of the home may affect home ventilation and therefore indoor radon concentration levels.

## 1. Introduction

### 1.1. Background

This project was undertaken to provide analytic support to the Community Uranium Exposure Journey to Healing Program (CUEJTH), which is sponsored by the Navajo Area Indian Health, Service located in Shiprock, New Mexico [1]. The Navajo Nation is located on the Colorado Plateau, a region which contains high levels of natural uranium in the soil and rocks [2,3]. A decay product of uranium is radon, a lung carcinogen, which has no color or odor and is able to diffuse from the soil into homes. Radon is a radioactive noble gas that is inert, colorless, odorless, denser than air, and soluble in water [4]. Radon concentration in the air is measured in units of radioactivity per volume of air, picoCuries per Liter (pCi/L) or Becquerels per cubic meter (Bq/m^3^), when using the standard international (SI) units [5].

The exposure to radon in non-occupational settings could potentially occur from naturally enriched soil with uranium or from anthropogenic hazardous waste, such as abandoned uranium mines (AUMs), or wastes from uranium mine and mill tailings [6]. According to the United States Environmental Protection Agency (US EPA), during the period between 1944 and 1986, approximately four million tons of uranium from the Navajo Nation was extracted, leaving behind over 500 abandoned uranium mines (AUMs). Thus, people who live near uranium mines may be exposed to greater levels of uranium and radon compared to communities who do not live near such sites [7]. Radon itself decays into radioactive progeny that are capable of binding to dust particles, creating a potential for inhalation exposure. Health effects associated with occupational exposure to uranium and its decay progeny, which includes radon, have been well established in previous epidemiologic studies [8,9,10,11,12,13]. In contrast, there are fewer studies of the health effects associated with radon exposure in non-occupational settings.

### 1.2. State-of-the-Art

Previous research has been published that have assessed home indoor radon concentration in various geographic regions and seasons, using either short- or long-term radon kits, along with capturing housing characteristics with home survey questionnaires [14,15,16,17,18]. Established predictors associated with home indoor radon concentration levels include housing characteristics, e.g., house type and type of foundation, indoor ventilation, air pressure differences, temperature, groundwater, and geological characteristics. In one related study, researchers examined building characteristics in association with indoor radon concentration levels, by using a multiple regression model [16]. Using available survey data obtained from questionnaires in Northern Italy during the time period September–November 2003, the authors reported that indoor radon concentration levels in buildings with direct contact to the ground were higher compared to buildings with a crawl space, 146 Bq/m^3^ and 111 Bq/m^3^, respectively. Similarly, buildings with stone wall structural materials were observed to have higher indoor radon concentration levels than buildings made of other materials, such as hollow brick, 161 Bq/m^3^ and 118 Bq/m^3^, respectively. In a second similar study, researchers in the US aimed to assess housing characteristics in association with home indoor radon levels [18]. This study was conducted during the time period March–May 2015, among randomly selected household participants utilizing short-term indoor radon kits, 3–5 days, and a home survey checklist. Notably, the authors observed that home foundations with a crawl space compared to home with direct slab on grade, had significantly lower levels, 29.8 Bq/m^3^ and 36.5 Bq/m^3^. This research contributes to previous studies by utilizing similar indoor radon assessment approaches in a geographical area predicted to have elevated home indoor radon concentration levels. More importantly, this study is unique, because it was designed and completed in collaboration with the Navajo Nation, which is rich in cultural language, traditions, and lifestyle.

### 1.3. Sources of Home Indoor Radon Exposure

The Navajo Reservation lies geologically in a region that contains higher concentrations of natural soil uranium [2,3,19]. Because natural soil uranium continuously undergoes radioactive decay, the uranium decay byproducts, radium and radon gas are constantly produced. The Environmental Protection Agency (EPA) developed a radon zone map across the U.S. using national survey housing radon data that did not include many tribal nations. This EPA radon zone map predicts that homes on the Navajo Nation may have estimated indoor radon levels between 74.0 to 148.0 Bq/m^3^ of radon gas. Individuals who live near uranium mines may also be potentially exposed to higher levels of radon compared to the general population [5,20]. A home built in this type of landscape, specifically upon soil with elevated levels of natural uranium concentration levels, is a potential main source of indoor radon exposure. Housing conditions, e.g., poorly sealed windows and doors, impact the overall air ventilation into and out of the house. Housing construction materials have been shown to contribute to elevated levels of indoor radon concentration levels. Historically, some homes on the Navajo Nation were built with uranium mill tailings waste materials, which could contribute to elevated levels of indoor radon concentrations [21,22]. Homes on the Navajo Nation include the hogan, a traditional type of home commonly constructed of wood and mud for the frame, round-shaped and often with a floor foundation made-up with the natural hard-packed earth soil. This particular type of traditional home construction built in this type of natural landscape necessitates a call to action to expand home indoor radon knowledge and assessment to Navajo Nation housing authorities and homeowners.

Environmental factors that include pressure differences inside and outside the home, temperature, and elevation, must also be taken into account when assessing home indoor radon levels. Lower pressures inside the home compared to the outside create a pressure vacuum in which air from the soil, which may contain radon gas, replaces air inside the home known as the stack effect [23,24,25]. Colder temperatures may also indirectly influence home indoor radon concentration levels. Seasons in which windows and doors are closed could may result in lower ventilation in the home, potentially elevating indoor radon levels. The ventilation rate at which outside air enters and leaves the home can be affected by domestic behaviors inside the home. In colder weather conditions, mechanical ventilation systems are often used to warm air inside the home, creating the stack effect [26]. Further, in colder seasons domestic behaviors, such as closing windows and doors, decrease the overall natural ventilations of air into and out of the home. As a result, there is the potential for radon gas to accumulate inside the home during colder seasons. Temperature and humidity affect the exhalation rate of radon from the soil surrounding the home, usually in the inverse direction. For instance, increased atmospheric pressure, rain, and snow reduce the exit of radon gas from the soil, thereby increasing the accumulation of radon gas in the soil. This may then result in the movement of radon gas from the soil inside the home [22]. As a consequence, this would potentially increase radon concentration in the home during the winter season. Combined with a closed housed system and increased soil radon concentrations in the winter, this would provide optimal conditions for elevated indoor radon concentrations.

Groundwater is also a source of potential radon gas when stored inside the home. Radon gas is able to dissolve in groundwater, which is a consequence of surrounding rock enriched with radium-226 such as near uranium ore bodies, often containing 238-U and radium-226 [22,27,28]. Groundwater compared to surface water has the potential to accumulate more radon gas, especially in geographic areas enriched with uranium [26,29]. When this enriched groundwater enters the home and is used, increasing the agitation and mixing of water through uses such as bathing and laundering, radon gas can be released via evaporation from the water into the air, potentially increasing inhalation exposure.

### 1.4. Health Effects Associated with Radon Exposure

The health effects of radon inhalation have been extensively studied. Much of this work is summarized in the sixth in a series of reports of National Academy of Sciences entitled The Biological Effects of Ionizing Radiation (BEIR). The BEIR VI report compares radon exposure levels between the occupational and residential setting, based on data from an indoor radon survey conducted by the Environmental Protection Agency. The BEIR VI committee estimated that residential indoor radon concentrations typically range from one-tenth to one-hundredth of the radon concentration observed in underground mines. The BEIR VI committee stated that the data suggest that indoor radon exposure in homes is associated with a small increase in lung cancer risk. Using indoor radon data from the National Residential Radon Survey conducted in the US, the BEIR VI committee estimated that among never-smokers, the number of lung cancer deaths due to radon was roughly 2100–2900 per year [30].

### 1.5. Environmental Protection Agency Home Indoor Radon Recommendation Levels

Concerns were raised about residential radon exposure during the 1980s when the Environmental Protection Agency (EPA) mandated a national residential radon survey (NRRS) of homes [31]. In the NRRS, the EPA reported in 1992 that the average annual radon concentration for U.S. homes was 48.1 Bq/m^3^, the average ambient annual radon level was 14.8 Bq/m^3^, and that roughly 6% of U.S. homes (~6 million homes) had annual average radon levels above the EPA action level of 148.0 Bq/m^3^. Recognizing these indoor radon estimates may not necessarily reflect homes on the Navajo Nation, which provides the motivation to conduct more indoor radon assessments in homes unique to the Navajo Nation, like the traditional hogan.

## 2. Materials and Methods

### 2.1. Ethics Approval and Consent to Participate

Two institutional review boards approved this project, the Navajo Nation Human Research Review Board (NNHRRB) (#NNR 14.185) and the University of Washington (UW) Human Subjects Division (#47652-EJ). The Navajo Nation Human Research Review Board (NNHRRB), established in 1996 on the Navajo Nation, was implemented to protect and ensure all research was done ethically in collaboration with the Navajo people [32]. Per NNHRRB protocol, an application was submitted, reviewed, and approved by the NNHRRB (#NNR-14.185), prior to the initiation of any research activities [33]. This board is led by a chairwoman, community members, health professionals, and scientists familiar with the Navajo Nation culture, language, and traditions. The NNHRRB mission states that research projects should promote the interests and visions of the Navajo people. 

**Consent for Publication:** This manuscript does not contain any individual person’s data in any form. Indoor radon data and home survey characteristics that were collected are provided in aggregate form. The approximate residence of CUEJTH participants within each Navajo Area Administrative Agency were dithered to protect the privacy of each individual. 

**Availability of Research Data and Materials:** The Navajo Nation Environmental Protection Agency Radon Program, located in Window Rock, AZ, maintains ownership for home indoor radon data on the Navajo Nation; and can be contacted at (928) 871-7863. Per Navajo Nation Human Research Review Board protocol, access to research data must be requested through the Navajo Nation Human Research Review Board located in Window Rock, AZ [32]. House images were purchased from iStock by Getty Images [34].

### 2.2. Design

The aim of the study was to collect indoor radon measurements from a sample of participants from the Community Uranium Exposure Journey to Healing (CUEJTH) program. The CUEJTH program was established to help address community concerns on the Navajo Nation regarding exposure to uranium, including decay products such as radon [35].

#### Setting and Sampling Strategy

This study was a home indoor radon survey conducted on a sample of CUEJTH participants (1) who indicated an interest to participate in research, (2) who are residents of the Navajo Nation, and (3) were residents in either the Chinle/Central, Shiprock/Northern, or the Crownpoint/Eastern agencies. A sample of indoor radon measurements previously measured across the Navajo Reservation during the time period 1997–2012 was provided by the Navajo Nation Air & Toxics Department Radon Program [36]. Based on this prior radon data, we recognized some differences across the Navajo Nation Agencies and allocated our samples according to this distribution. We aimed to contact 25 participants within each of the three agencies, for a total of 75 indoor radon measurements. Participants were contacted by telephone using two standardized scripts, a recruitment script and an informed oral consent script. All research scripts were translated into the Navajo language and approved by the Navajo Nation Human Research Review Board, to ensure materials were culturally appropriate.

### 2.3. Data Collection

#### 2.3.1. Home Characteristic Assessment Survey

**Figure 1:**
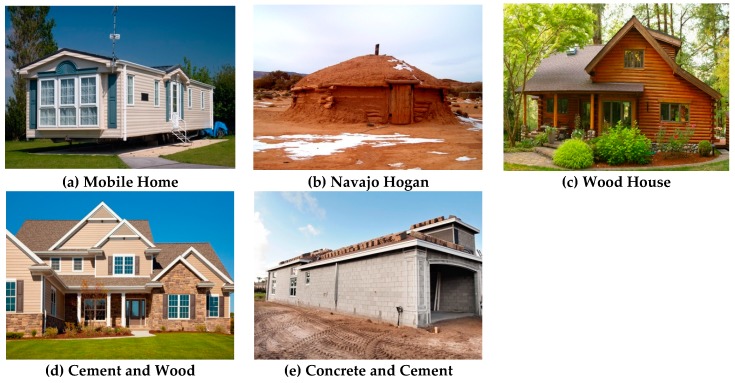
Five House Types.

Each home geolocation was recorded using a handheld GPS device, GARMIN eTrex 20 [37]. The local chapter and Navajo Nation Agency where the home was located were also recorded. Home characteristics and structural details were captured using a standardized checklist (As detailed in Supplementary material, Assessing Indoor Radon Exposure on the Navajo Nation Home Characteristic Checklist). Descriptions of the home were recorded as hogan, mobile, modular, rental, or other. The structural make-up of the home was categorized as concrete, brick or block, wood, earth/dirt, or other. Home descriptions and structural characteristics were then collapsed into five house types: mobile, hogan, wood, cement and wood, or concrete and cement (Figure 1a–e). These house images were not taken as part of this project; however, are intended to provide some structural design characteristics for each house [34]. The number of floors and approximate age of the home were also recorded. Structural components of the rooms in which radon kits were placed, e.g., concrete, brick or block, wood, earth/dirt, or other materials was noted. Using the checklist, the approximate fraction of each type of material making up the structural component of each testing room was recorded. Information regarding crawl spaces and type of materials surrounding it were documented. Using the checklist, each crawl space was assessed in terms of how much it was enclosed as either all, part, or none. The type of construction materials used to enclose the crawl space were categorized as: concrete, brick or block, vinyl, metal, or other. The type of foundation was recorded as either dirt, concrete, or wood. The overall condition of the foundation was also noted as either good, cracks, or other. Home ventilation was assessed by visually observing how tightly sealed doors and windows appeared, along with noting the operational condition of any home exhaust systems. Homes were categorized as either loose, intermediate, or tightly sealed. Floor characteristics were checked to note if any of the bottom floor exposed earth and the overall condition of the floor, e.g., visible cracks. Any visible cracks were categorized in size with additional notations on whether the cracks were even, uneven, vertical, or horizontal. Home exhaust fans were checked in terms of usage, diameter size, and functionality. Descriptions of home heating systems were recorded, e.g., woodstoves or fireplaces. Drinking water source and storage were obtained because water is a potential source of indoor radon exposure, if collected from water wells with elevated levels of uranium. Ceiling surface conditions, such as opening and cracks, were noted. 

#### 2.3.2. Indoor Radon Exposure Assessment

Short-term radon kits manufactured by Air Chek, Inc. were used following closed house condition per AirChek instructions for a minimum of 3 days and not more than 7 days [38]. Each home was measured only once, with one test kit placed in the living room, one in the bedroom, basement if applicable, and one additional radon kit for quality control with every tenth radon sample. The kits were analyzed by the Air Chek, Inc. Laboratory. The lower limit of detection (LOD) for Air Check indoor radon test kits in dry climates is 7.4 Bq/m^3^ [39]. Results below the LOD were returned as <11.1 Bq/m^3^. Using an accepted method, radon measurements lower than the LOD were replaced by taking the LOD and dividing by the square root of two (LOD/sqrt 2). Basic descriptive analyses were calculated for mean indoor radon concentration levels and log-transformed. Log-transformed radon data was evaluated by graphing on a histogram and evaluating using the Shapiro–Wilk test of normality. Following this observation, the geometric mean (GM) and geometric standard deviation (GSD) were computed.

#### 2.3.3. Temperature and Elevation Data

Historical temperature data during the home indoor radon testing period were obtained using two on-line weather data resources, “Weather Underground (WU)” and “The Weather Channel” [40,41]. Weekly temperature averages were available from both weather data resources, and used as the temperature value during household radon testing. Elevation data for each home were ascertained using a handheld GPS Garmin eTrex20 device. It is well known at higher elevations that temperature decreases at a uniform rate, up to 39,000 feet (11,887.2 m), which is known as the adiabatic lapse rate [42]. In dry conditions, this uniform rate is equal to 5.5°/1000 feet (304.8 m) and in humid conditions the rate is 3.5°/1000 feet (304.8 m) [42]. 

## 3. Results

### 3.1. CUEJTH Participants

A total of 969 participants were registered with the CUEJTH program with a mean age of 59.1 years (SD = 14.9). Over one-half of participants lived in New Mexico, approximately one-third in Arizona, and the remainder in Utah. By agency, over one-half of CUEJTH participants lived in the Crownpoint/Eastern Agency (Table 1). 

Among the 969 registered participants, 329 participants filled out research forms indicating a willingness to be contacted. A total of 62 participants were excluded from the baseline recruitment sample based on the following reasons: (1) deceased, (2) no current contact information, (3) residence off the Navajo Nation, (4) home in the Western or Ft. Defiance Agencies and (5) had the same residence as another CUEJTH participant. The “follow-up” sample consisted of 267 participants. From this follow-up group, 218 potential participants were excluded from the analytic sample for the following reasons, (1) no response, (2) Navajo translation preferred, (3) deceased, (4) no current contact information, (5) no answer or voicemail set up for working mobile numbers, (6) home visits cancelled by participant or due to weather, (7) participant too busy or not interested, (8) no phone number, (9) lived with other CUEJTH participant or home previously tested, (10) mobile service unavailable, and (11) participant current residence outside approved study agency.

### 3.2. Analytic Sample 

**Figure 2:**
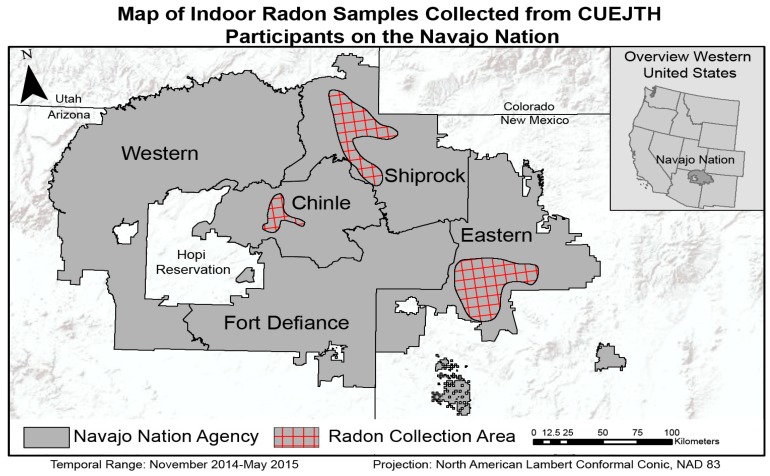
Map of Indoor Radon Samples Collected from CUEJTH Participants (red checkered areas) on the Navajo Nation (Sampling was only conducted in three Navajo Nation Agencies: Shiprock, Chinle, and Eastern).

The analytic sample consisted of 49 participants who were successfully contacted and completed a home indoor radon assessment. These participants were distributed across the three approved agencies as follows; frequency (percentage): (1) Shiprock/Northern, *n* = 22 (44.9%), (2) Crownpoint/Eastern, *n* = 23 (46.9%), and (3) Chinle, *n* = 4 (8.2%). Two individuals among the 49 participants owned and resided in two homes and requested that both their homes be tested. Including these two additional households brought the total analytic sample for households to 51, compared to 49 analytic samples for participants. The approximate residence of CUEJTH Participants who completed a home indoor radon assessment is mapped in Figure 2 according to their respective agency of residence on the Navajo Nation (dithered to protect identity) [43].

### 3.3. Home Assessment Characteristics

#### 3.3.1. Crawl Space

Table 2 provides a description of the overall basic house characteristics for certain variables thought to be associated with indoor radon concentration levels. Crawl spaces were observed in all 12 mobile homes (100%) surveyed in this study. Other home categories with observed crawl spaces were as follows: one of two hogans (50%); 11 of 27 wood homes (41%); and three of eight concrete and cement homes (38%). In contrast, no homes made of cement and wood were observed to have a crawl space.

#### 3.3.2. Cracked or Shifted Doors

Information was obtained on airflow or light shining through doors, usually as a result of cracked or shifted doors. For example, over time, some wood doors may have cracked or possibly shifted, due to a shift in the foundation of the home. As a result, airflow could be felt by standing next to a shifted door, merely by observing light shining through cracks of door side linings. Only two categories of home were observed to have cracked or shifted doors, mobile homes (25%) and wood homes (22%). The three remaining home categories were not observed to have cracks or shifts in their doors.

##### Home Exhaust Fans

Participants were asked if there was a main exhaust fan in the home. This did not include ventilation fans reported or observed in the bathrooms, laundry rooms, or above ovens. Only two home categories were observed to have some type of main exhaust fan, mobile (17%) and wood (11%) homes. The three remaining home categories did not have a main exhaust fan.

#### 3.3.3. Visible Cracks in the Floor

The two hogans surveyed were the only homes in which no visible cracks were reported or observed in the floor. Otherwise, almost half of each of the remaining home categories either reported or were observed to have visible cracks in the floors as follows: mobile (42%); wood (56%); mix “cement and wood” homes (50%); and mix “concrete and cement” homes (63%).

##### Woodstoves

Woodstoves were reported and observed in all home categories. Mix “cement and wood” and mobile homes were two home categories least observed to have a wood stove compared to other homes, 50% and 75%, respectively. Wood homes almost all had a woodstove installed, 93%, while the remaining two home categories, hogan and mix “concrete and cement”, all had woodstoves installed. 

#### 3.3.4. Ceiling Openings or Cracks

In each home category, ceiling cracks and openings were observed in at least half or more of each home. Hogans and mix “cement and wood” homes each had at least 50% openings or cracks in their ceilings. Mobile homes and wood homes were observed to have ceilings with openings or cracks, 58% and 59%, respectively. Mix “concrete and cement” homes had the largest proportion of ceiling openings and cracks, 75%, compared to other home categories.

### 3.4. Mean Indoor Radon Concentration Results 

Overall, 110 indoor radon kit measurements were obtained among the 51 homes sampled; 51 living room measurements, 47 bedroom measurements, two basement measurements; and 10 quality control measurements. All indoor radon concentrations were measured in picocurie/liter (pCi/L) and converted into SI units becquerels per cubic meter (Bq/m^3^). The correlation coefficient (r) between living and bedroom indoor radon concentration comparing non-log transformed levels was r = 0.98 (S.E. = 0.03) and the correlation coefficient comparing the log-transformed data was 0.95 (S.E. = 0.05). Based on these correlation results, all home indoor radon measurements were averaged to compute a mean indoor home radon concentration.

Mobile homes had an overall geometric mean (GM) indoor radon concentration of 20.6 Bq/m^3^ and a geometric standard deviation (GSD) of 2.6, which was the lowest observed indoor radon concentration by home type (Table 3). Hogan homes had an overall geometric mean indoor radon concentration of 74.0 Bq/m^3^ (GSD = 1.0). Homes constructed primarily of wood had an overall geometric mean indoor radon concentration of 43.6 Bq/m^3^ (GSD = 2.5). Homes with an observed mixture of cement and wood, referred to as mix cement and wood had an overall geometric mean indoor radon concentration of 82.6 Bq/m^3^ (GSD = 1.0). The highest mean indoor radon concentration was observed in homes constructed mainly of cement and concrete, with a geometric mean of 96.1 Bq/m^3^ (GSD = 1.6).

**Figure 3:**
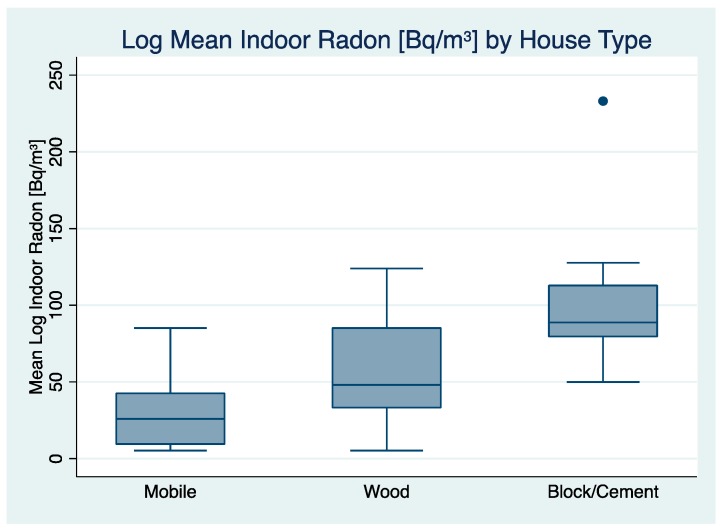
Log Mean Indoor Radon Concentration [Bq/m^3^] by House Type (Hogan and Mix “Cement/Wood” houses not included due to small sample size).

Notably, 11% of wood homes and 25% of mobile homes measured below the LOD. The remaining three home categories did not have indoor radon measurements below the LOD. Moreover, 13% of concrete and cement homes were observed to have indoor radon concentrations above 4.0 pCi/L (148.0 Bq/m^3^). Figure 3 provides a frequency distribution of mean radon concentration [Bq/m^3^] by house type.

### 3.5. Mean Indoor Radon Concentration by Ventilation

Ventilation was assessed by categorizing homes that were built directly on grade surfaces, compared to homes not built directly on grade surfaces that had a crawl space. The radon concentration levels were then compared between these two categories providing one approach in assessing how ventilation is associated with the observed indoor radon concentration levels in the dataset. This comparison is shown in Table 4. 

A two-sample t-test of the arithmetic mean indoor radon concentration levels by crawl space was assessed using the above data. The H_0_: B1 = B2 = 0; HA: B1 = B2 ≠ 0. Based on the two-sample t-test results, there is strong evidence that the arithmetic mean indoor radon concentration across the homes with crawl space, compared to homes with no crawl space, are not equal (*p* = 0.002).

### 3.6. Mean Indoor Radon Concentration by Agency

In Table 5 by agency, the overall AM of mean indoor radon concentration was 60.5 Bq/m^3^ with a SD of 42.7. Highest to lowest AM (SD) of mean indoor radon concentration for each agency was as follows: Shiprock/Northern, 74.4 (47.7), Chinle, 53.2 (20.8), and Crownpoint/Eastern 48.5 (37.0).

### 3.7. Association of Indoor Radon with Temperature and Elevation

The log mean indoor radon concentration showed a small decrease in mean home indoor radon concentration at higher temperatures (*r* = −0.10; S.E. = 0.14). This was a non-significant trend, as the data collection period occurred primarily during the winter season months −1.1 °C to 15.6 °C. The log mean home indoor radon concentration values by elevation (meters) showed a negative relationship (*r* = −0.40; S.E. = 0.13).

## 4. Discussion

This home survey is unique, because it is one of the first studies on the Navajo Nation to explore the factors associated with home indoor radon exposures. A vast number of occupational epidemiological studies have been published on the association between radon progeny exposure and risk of lung cancer among Navajo uranium miners; however, very few studies in the US, including the Navajo Nation, explored radon exposure outside the workplace in a community setting. The findings in this survey correlate with other studies regarding the association between home ventilation, housing characteristics, and environmental factors with home indoor radon exposure levels. However, home indoor radon estimate maps, such as the EPA radon zone map, historically did not include home indoor radon levels on the Navajo Nation, nor account for traditional style hogan homes on the Navajo Nation. Some home structures on the Navajo Nation were built with uranium mill waste materials that were left abandoned, which provides motivation to assess homes for indoor radon levels across the Navajo Nation.

### 4.1. Basic Ventilation Characteristics by House Type

Home substructures that sit above the soil, e.g., mobile homes, have a built-in crawl space that may influence soil radon gas entry into a home [26]. In this study, all observed mobile homes sat above the soil surface and had crawl spaces compared to other house types. This crawl space between the soil and the foundation of the home, uncouples the ventilation inside the home from the soil surface, which may account for the observed lowest indoor radon concentration levels observed in this house type. This observation aligns with previously published literature reporting how substructure foundations elevated above soil surface, especially with ventilated crawl spaces, are less likely to have high indoor radon concentration levels, compared to homes built directly on top of soil. In comparison, two types of homes with higher levels of indoor radon concentrations had either none or less than half of the homes with crawl spaces. Thus, a home foundation that sits directly on soil surfaces couples inside ventilation the home with the soil, which is known to influence indoor radon concentration levels.

Visible cracks in the floor were observed in all house types except hogans. These floor conditions could impact the amount of radon gas passing into the home from the soil. Only two hogans were measured for indoor radon levels and combined, the two hogans were observed to have higher levels of indoor radon concentration levels. This observation may have been impacted by the construction of these traditional local home types. Traditionally, hogans are constructed of local soil and materials, and historically, some materials were used from abandoned uranium mines [44].

In comparison to traditional hogans, homes manufactured in an industry standardized method, e.g., mobile homes, may share common wear and tear complications. Common observations in all mobile homes surveyed included cracked or shifted doors, exhaust fans, and openings or cracks in the ceiling. These home characteristics may affect the infiltration rate of any radon gas into all mobile homes similarly. Homes categorized as “wood” were the only other house type observed to have cracked or shifted doors. Mobile and wood house types had home exhaust fans, while the other house types did not. Ceiling cracks and openings were observed in all house types. Cracked and shifted doors, along with home exhaust fans, may also contribute to the lowest indoor radon concentration levels observed in mobile homes.

### 4.2. Averaging Mean Indoor Radon Concentration Results

Living and bedroom radon concentration results indicated little variation in rooms above ground. Only two homes had basements and both basements had higher levels of radon concentration, which is consistent with current literature. These observations provided justification for providing one average radon concentration level as the radon exposure for each home.

### 4.3. Mean Indoor Radon Concentration Results by House Type

It has been previously documented that house materials can impact the level of indoor radon concentrations [45]. Mobile homes had the lowest observed indoor radon concentration levels compared to the other house types. Mobile homes are manufactured homes and range in size from single wide to double wide homes, and can be longer than 40 feet in length and wider than 8 feet [46]. Because mobile homes are usually mounted on wheels and positioned above the surface, as was observed in all mobile homes for this study, there is no direct contact between the home foundation and ground surface. This creates a crawl space, which is usually enclosed with various materials, such as vinyl siding or bricks [46]. This space underneath the home creates an opportunity for air infiltration and ventilation, to impact any potential radon gas emitted from the underground soil to potentially escape from underneath the home and accumulate inside. Therefore, this limits the amount of potential radon gas to enter the home, leading to lower indoor radon concentrations, as was observed in mobile homes.

Homes constructed primarily of wood had the next lowest observed indoor radon concentration levels. Homes built with wood in the structural component usually have deep cavities that require certain amounts of insulation to be layered in between the walls [47]. Historically, the spaces in these cavities provided natural infiltration and exfiltration of indoor air in the U.S. [48] Over time, in the 1980s and 1990s, homes were constructed in a more enclosed manner, with higher insulation aimed at energy efficiency. In Florida, a comparison between wood frame homes versus concrete block homes stated that wood frame homes constructed with lumber were more poorly designed and did not offer much durability compared to more durable materials, such as concrete [48]. Depending on the insulation material and the amount of spacing between lumber used in the walls, this could create an environment in which any potential indoor radon gas may escape. Based on this knowledge of lumber use in home construction, it is probable to observe lower mean indoor radon concentration, as observed in these wood-framed homes.

Homes with the third highest mean indoor radon concentration for this project were hogans. The hogan is a traditional type of Navajo home used for traditional ceremonies and often constructed with wood, bark, and mud [49]. Typically, hogans are round-shaped, with an opening at the top with one opening doorway, always facing the east to greet the morning sunrise. The opening at the top is to allow smoke from a wood stove to escape out. The foundation of hogans usually consist of a hard-packed earthen floor, which could potentially increase the accumulation of radon gas emanating from the soil into the hogan [50]. Based on this structural design, any potential indoor radon gas could potentially escape through any openings in the structural walls not sealed, or through openings at the top where smoke exits the home.

Homes constructed with a mixture of wood and cement had the second highest observed indoor radon concentration levels. Considering that homes built with cement are more durable and potentially more enclosed, this provides insight into the observed mean indoor radon concentration. Combined with lumber, these homes, if potentially more insulated, may potentially have higher indoor radon concentrations compared to mobile homes, or homes made up of only lumber, as was observed in this study.

Homes constructed primarily of concrete and cement in this study had the highest observed mean indoor radon concentration levels. Homes primarily constructed with concrete and cement were observed to have these materials in both the foundation and structural walls. Such homes usually have a more solid structural component and are commonly used as structural materials, because they provide greater resistance to certain weather conditions, such as heavy winds and rain, according to the National Association of Home Builders [51]. Concrete and stones can also naturally contain radium, and homes built mainly with concrete with reduced levels of fresh air entering the home have been observed to have problems associated with radon concentrations [52]. Homes with a more solid foundation and positioned above the ground surface, and in areas with potentially higher soil uranium concentrations, could potentially have elevated indoor radon levels, as observed in these homes. Further, if there are no cracks in the concrete slab foundation, then a home may be considered more tightly sealed, which would then allow any potential radon gas beneath the home to accumulate inside the home.

### 4.4. Mean Indoor Radon Concentration by Temperature

The indoor radon concentrations were primarily collected during the winter season, extending from November 2014 through early May 2015. The flow of radon gas into the home is affected by the amount of radon gas that can enter the home, such as through cracks in the foundation or through spaces around pipes. This movement of gas is enhanced by pressure difference inside and outside the home. Temperature differences (i.e., indoor heating) can create a lower pressure environment inside the home compared to a higher pressure outside the home, which is known as the stack effect [53]. This then favors the movement of radon gas entering the home.

In this survey with CUEJTH participants, we collected most of the indoor radon measurements during the winter season. During colder conditions, home domestic behavior is also different to periods of warmer temperatures. For example, during colder conditions, homeowners tend to keep windows and doors closed. Further, in the winter, it may snow or rain more frequently, and these types of moisture naturally create a blanket over the soil, which negatively impacts any soil emanation. This reduced level of soil breathing may influence any potential radon gas in the soil to move into the home. Taking this into consideration, we expected to observe higher indoor radon concentrations at colder temperatures, compared to lower indoor radon concentrations at higher temperatures. This change in temperature between the outside and inside of the home affects the pressure difference, and this then draws radon gas into the home. Because we restricted our collection period to mainly the winter season, we did not observe a significant correlation between home indoor radon concentrations and temperature during testing. This is most likely due to our short testing period, restricted to the winter season.

### 4.5. Mean Indoor Radon Concentration by Elevation

The log transformed mean home indoor radon concentration plotted by elevation (meters) showed a negative relationship. At higher altitudes, it is known that temperature decreases uniformly, which is known as the lapse rate [42]. Up to 10 km, this lapse rate is equal to 6.5 °C/1000 m. Based on this knowledge, we would expect the mean indoor radon concentrations to increase at higher elevations, as is observed with lower temperatures. Further, these indoor radon measurements were collected during the winter season, a time when a home is more tightly sealed by closing windows and doors to keep warm air inside the home. These conditions also increase the stack effect inside the home; the heating effect when warm air inside the home rises, creating a vacuum inside the home that is replaced by outside air moving into the home [23]. Therefore, this indoor environment created by the cold temperature and domestic behaviors would potentially favor the accumulation of any indoor radon gas.

### 4.6. Limitations

#### 4.6.1. Barriers to Household Participation in a Rural Area

##### Response Rate

The low household response rate of 19% was due to several factors. Almost one-third of potential participants did not respond; primarily due to unanswered messages left for this group (29%). Another factor was related to no current contact information for some participants (21%). Further, if these individuals did not recently visit their respective local Indian Health Service clinics, then this would account for a lack of current information recorded in electronic health records. A certain proportion of participants, 14%, also stated that they were not interested or too busy. Another group simply did not respond to voicemail message or other messages (12%).

The Navajo Nation access to broadband cellular phone usage is lower than the national average, 53% vs. 98%, as reported by the Navajo Times in April 2013 [54]. This illustrates the proportion of residents living on the Navajo Nation without access to cell phones or Internet services. Access to land line telephones is higher on the Navajo Nation, 68%, but still lower than the national rate of 98% [54]. Compared to other tribal reservations and the U.S., the Navajo Nation is lagging behind in cellular phone and Internet access. Thus, limited access to cellular phone and land-use telephone services may partially account for the proportion of residents who were not contacted, due to no current contact information, or who may not have responded to voice mail messages.

The indoor radon survey took place primarily during the winter season, which may have resulted in two potential limitations. One limitation is related to how radon was assessed primarily during the colder season. There is seasonal variation of indoor radon concentration; however, this study was primarily restricted to the winter season, a time when indoor radon concentrations have been observed to be higher compared to lower levels in the summer [15]. A second potential limitation conducting home assessments in the winter is that many residents were unavailable at home. As a result, many potential participants indicated that their personal lives were too busy or unpredictable to state whether they would be available for two home visits. Others simply stated an appreciation for the project, but overall, were not interested in their home being tested for indoor radon. Scheduled visits were also cancelled due to unforeseen family emergencies, where the participant would be unavailable at home, or if the weather did not permit a home visit.

##### Non-Response Bias

The overall household response rate was 19% in this indoor survey, which may reflect several factors. One factor is the low availability of cellular phone usage on the Navajo Nation. Nearly half of the follow-up sample participants who were eligible had no first response or had any recent contact information. However, smaller proportions of these eligible participants stated that they had no time or were too busy, even though they may have been interested in participating. All efforts were made in attempting to accommodate each participant with home visits; however, if the participant stated no interest, then this decision was respected. Given these observed barriers, a household response rate of 19% is respectable for home visit surveys conducted during the winter season, limited cellular coverage, and a unique traditional language. Interestingly, only a small proportion of participants who did not participate were not included because they may have preferred the Navajo language at the first phone call. Attempts were made in advance with each phone call to provide Navajo language translation.

##### Selection Bias

To reduce potential selection bias, CUEJTH participants were selected in a systematic approach. First, participants were only eligible if they filled out a form indicating a willingness to be contacted. Second, participants had to have homes located on the Navajo Nation. Third, participants had to have a residence located in one of the three approved agencies. Further, eligible participants were categorized within their respective agencies, chapter regions and then randomized.

## 5. Conclusions

This home indoor radon survey with participants from the CUEJTH program provided updated indoor radon measurements for homes on the Navajo Nation. Of the homes observed in this study, 13% of concrete and cement homes were observed to have indoor radon concentrations above the EPA action limit of 4.0 pCi/L (148.0 Bq/m^3^). This observation is higher than the number of homes in the U.S. reported to be above the EPA action limit. The home survey was developed, reviewed, and approved by members of the Navajo Nation Human Research Review Board, made up of researchers and community members versed in the Navajo language, culture, and traditions. Scripts were translated into the Navajo language and Navajo translation was offered for participants who preferred the Navajo language.

House type characteristics and type of foundations have been examined in recent studies using survey questionnaires, as was done in this study [14,15,16,18]. In these environmental research studies, the authors used questionnaires to collect data on the type of house, building characteristics, cracks in the foundation, use of groundwater, and ventilation factors. In one study, authors reported that radon levels were higher in buildings constructed of stone walls, compared to buildings constructed with other types of materials, such as hollow brick, 161 [Bq/m^3^] and 118 [Bq/m^3^], respectively [16]. In a second related study, researchers in the US noted that home foundations with a crawl space, compared to homes with direct ground contact, had significantly lower indoor radon levels, 29.8 Bq/m^3^ and 36.5 Bq/m^3^ [18]. These previous findings coincide with results in this study, because mobile homes were observed to have the lowest mean indoor radon concentration levels, while homes constructed primarily with concrete and cement had the highest mean indoor radon concentration levels.

House ventilation characteristics have also been assessed in recent studies by characterizing the usage of ventilation systems and infiltration factors [14,15]. In this study, ventilation was assessed by comparing indoor radon levels in homes with a crawl space to homes without a crawl space. In this study, mobile homes were observed to have the lowest mean indoor radon concentration levels; an observation not surprising considering each mobile home was set on various types of support systems (e.g., cinder blocks), creating a crawl space between the floor of the home and the soil top. Further, crawl spaces enclosed with vinyl coverings provide a less tightly sealed space, allowing any potential radon gas emanating from the soil underneath the home to exit through the crawl space; instead of radon gas entering the bottom of the home if the crawl space was tightly sealed.

The indoor radon concentration levels in this study agree with published information on the Navajo Nation. In regard to indoor radon concentration levels, the US EPA Map of Radon Zones categorizes the Navajo Nation as a Zone 2 region, which are areas with predicted average indoor radon levels ranging from 2.0 to 4.0 pCi/L (74.0 Bq/m^3^ to 148.0 Bq/m^3^). The southwestern region of the US is also known to have elevated levels of natural uranium in the soil. This observation, along with the historical legacy of abandoned uranium waste piles throughout the Navajo Nation, creates an environment with potentially higher levels of indoor radon concentrations. This environmental concern has been raised in other communities surrounded by uranium mining and milling outside the Navajo Nation. Currently, the EPA radon zone map does not allow one to predict home indoor radon levels above the EPA action limit as published. This type of map could be developed using the findings of this study and local geographic information from the Navajo Nation.

The findings of this indoor radon survey have a few potential public health implications for homeowners on the Navajo Nation. First and foremost, radon education and testing on the Navajo Nation must continue through the Navajo Nation Environmental Protection Agency Radon Program. Second, homeowners must continue to be informed about the importance of adequate ventilation throughout the home, especially during colder temperatures when homes tend to be more tightly enclosed, i.e., with windows and door shut. Currently, no public housing databases listing indoor radon levels for distinctive homes on the Navajo Nation, e.g., the hogan, compared to standardized homes exist. Third, the Navajo Nation is a geographical region historically impacted by abandoned uranium mines and natural elevated levels of soil uranium concentrations, which are two environmental indicators previously known to be associated with higher concentrations of indoor radon. Therefore, community education programs, such as the CUEJTH program, can benefit from the findings in this study about home type, and must continue to be supported to provide communities with education and basic training surrounding uranium, such as testing homes for indoor radon levels and mitigating, if necessary [55].

## Figures and Tables

**Table 1:** Community Uranium Exposure Journey to Healing Program (CUEJTH) Population and Navajo Residents Proportion by Navajo Nation Agency.

		2010–2013 CUEJTH Population Proportion *n* = 969 (%)	* 2010 Proportion of Navajo Residents *n* = 173,637 (%)
Agency	Chinle	3.3	16.0
	Crownpoint/Eastern	57.7	19.2
	Fort Defiance	3.3	25.3
	Shiprock	10.7	17.8
	Tuba City/Western	23.7	21.7
	Missing	1.3	
		100.0	100.0

* 2010 US Census.

**Table 2:** Basic Ventilation Characteristics by House Type.

Ventilation Characteristics	Mobile	Hogan	Wood	Cement/Wood	Concrete/Cement
	*n* = 12	*n* = 2	*n* = 27	*n* = 2	*n* = 8
	(%)	(%)	(%)	(%)	(%)
Crawl Space	100	50	41	0	38
Door Cracks/Shifted	25	0	22	0	0
Exhaust Fan	17	0	11	0	0
Visible Cracks in the Floor	42	0	56	50	63
Woodstove	75	100	93	50	100
Ceiling Openings/Cracks	58	50	59	50	75

**Table 3:** Mean Indoor Radon Concentration [Bq/m^3^] by House Type.

	N	GM	GSD	AM	SD	Min	Max
All Homes	51	43.3	2.6	60.5	42.7	5.2	233.1
Mobile	12	20.6	2.6	29.0	22.9	5.2	85.1
Hogan	2	74.0	1.0	74.0	0.0	74.0	74.0
Wood	27	43.6	2.5	58.6	36.0	5.2	124.0
Mix (Cement/Wood)	2	82.6	1.0	82.6	3.5	80.2	85.1
Concrete/Cement	8	96.1	1.6	105.7	55.8	50.0	233.1

**Table 4:** Mean Indoor Radon Concentration [Bq/m^3^] Level by Navajo Nation Agency.

Crawl Space	Observations	AM	SD	Min	Max
Yes	27	43.9	31.1	5.2	107.3
No	24	79.3	46.6	5.2	233.1

**Table 5:** Mean Indoor Radon Concentration [Bq/m^3^] Levels by Navajo Nation Agency.

Agency	N	GM	GSD	AM	95%CI LL	95% CI UL	SD	Min	Max
All	51.0	43.3	2.6	60.5	48.6	72.5	42.7	5.2	233.1
Shiprock/Northern	23.0	62.0	1.9	74.4	53.8	95.1	47.7	22.2	233.1
Crownpoint/Eastern	24.0	30.0	3.2	48.5	32.9	64.1	37.0	5.2	127.7
Chinle	4.0	50.3	1.5	53.2	20.1	86.3	20.8	31.5	81.4

## Supplementary Materials

The following are available online at https://www.mdpi.com/1660-4601/17/8/2813/s1, Assessing Indoor Radon Exposure on the Navajo Nation Home Characteristic Checklist.
