# Supplementary material for: Assessing the Impact of Housing Features and Environmental Factors on Home Indoor Radon Concentration Levels on the Navajo Nation
Source: Int J Environ Res Public Health. 2020 Apr 19;17(8):2813. doi: 10.3390/ijerph17082813 (PMC7215699; doi:10.3390/ijerph17082813)
Supplement: Supplementary file 1 [file ijerph-17-02813-s001.pdf]

SCHOOL OF PUBLIC HEALTH
UNIVERSITY of WASHINGTON

Assessing Indoor Radon Exposure on the Navajo Nation
Home Characteristic Checklist

The purpose of this home checklist is to geocode the location of homes and record basic house information from participants in the study.

Participant Number: ____

Location of home: Latitude: ________________________________

Longitude: ______________________________

Chapter/Agency: ____

GPS Device (model) ____

Researcher Name: ____

Date(s) of home visit 1st visit: ______/_____/______
Month Day Year

Time began: ________________ am/pm

Time finished: ________________ am/pm

Reminder of scheduled visit ______/_____/______
Month Day Year

Method: email/phone call

2nd visit: ______/_____/______
Month Day Year

Time began: ________________ am/pm

Time finished: ________________ am/pm

Reminder of scheduled visit ______/_____/______
Month Day Year

Method: email/phone call

Assessing Indoor Radon Exposure on the Navajo Nation
Home checklist

A. Structure of Home

1. Which of the following best describes the home?
   - (01) Hogan ________
   - (02) Mobile ________
   - (03) Modular ________
   - (04) Rental ________
   - (05) Other: ____

2. Which of the following best describes the home lived in most of the time? (if summer and winter homes, then pick winter home)
   - (01) Hogan ________
   - (02) Mobile ________
   - (03) Modular ________
   - (04) Rental ________
   - (05) Other: ____
   - (06) Unknown: ________

3. How many floors are in this home?
   - (01) 1 story ________
   - (02) 2 story ________
   - (03) Other: ____

4. Where do you spend most of the time in this home (other than where you sleep)?
   - (01) 1 story ________
   - (02) 2 story ________
   - (03) Other: ____
   - (04) Unknown ________

5. Do you know what when your home was built?
   - (01) Month/year ________
   - (02) Unknown ________

6. About how old is this home (where time is spent the most)?
   - (01) Less than year ________
   - (02) 1-5 years old ________
   - (03) 6-10 years old ________
   - (04) 11-20 years old ________
   - (05) >20 years old ________
   - (06) Unknown ________

7. How long have you lived in this home?
   - (01) ________ months
   - (02) ________ years

8. Which of the following best describes most of the structural component of the home?
   - (01) Concrete ________
   - (02) Brick or Block ________
   - (03) Wood ________
   - (04) Earth/dirt ________
   - (05) Other: ____

9. Which of the following best describes most of the structural component of the rooms in which radon test kits are placed?

   What fraction (square footage of home)?

   - (01) Concrete ________ (a) 25% (b) 50% (c) 75% (d) >75%
   - (02) Brick or Block ________ (a) 25% (b) 50% (c) 75% (d) >75%
   - (03) Wood ________ (a) 25% (b) 50% (c) 75% (d) >75%
   - (04) Earth/dirt ________ (a) 25% (b) 50% (c) 75% (d) >75%
   - (05) Other: ________ (a) 25% (b) 50% (c) 75% (d) >75%

10. Is any part of the home over a crawl space? (space between the ground and the floor structure of the home)
    - (01) Yes ________
    - (02) No ________
    - (03) Other: ____

11. How much of the crawl space is enclosed?

			What fraction?
(01)	All	________	(a) 25% (b) 50% (c) 75% (d) >75%
(02)	Part	________	(a) 25% (b) 50% (c) 75% (d) >75%
(03)	None	________	(a) 25% (b) 50% (c) 75% (d) >75%
(04)	Other:	________	

12. Which of the following best describes the construction material of most of the outside of the crawl space?

			What fraction?
(01)	Concrete	________	(a) 25% (b) 50% (c) 75% (d) >75%
(02)	Brick or Block	________	(a) 25% (b) 50% (c) 75% (d) >75%
(03)	Vinyl	________	(a) 25% (b) 50% (c) 75% (d) >75%
(04)	Metal	________	(a) 25% (b) 50% (c) 75% (d) >75%
(05)	Other:	________	(a) 25% (b) 50% (c) 75% (d) >75%

13. What type of foundation is the majority of the home foundation constructed of?

(01)	Dirt	________
(02)	Concrete	________
(03)	Wood	________

14. Overall condition of doors
    (01) Good
    (02) Cracks
    (03) Other ____

15. How tightly sealed is the house?

Loosely | Intermediate | Tightly

Loosely: Drafts of air, worn door strips, visible light through door
Intermediate: Little or no air draft, door strips intact, limited visibility of light through door
Tightly: No air draft, door strips near new, no visible light through door

(01) Doors

Front — Loosely | Intermediate | Tightly

Rear — Loosely | Intermediate | Tightly

Other — Loosely | Intermediate | Tightly

(02) Windows

Location:_________ — Loosely | Intermediate | Tightly

Location:_________ — Loosely | Intermediate | Tightly

Overall:_________ — Loosely | Intermediate | Tightly

Skylight ________ — Loosely | Intermediate | Tightly

(03) Sliding door ________ — Loosely | Intermediate | Tightly

B. Characteristics of floor

16. Is any part of bottom floor exposed earth?

(01) Yes ________ (a) 25% (b) 50% (c) 75% (d) >75%

(02) No ________

(03) Other: ____

17. What is the overall condition of the floor?

	Material		Condition
(01)	Concrete slab	________	(a) Smooth/Solid (b) Visible cracks
(02)	Dirt	________	(a) Smooth/Solid (b) Visible cracks
(03)	Wood	________	(a) Smooth/Solid (b) Visible cracks
(04)	Other	________________________________	

18. Description of visible cracks in the floor:

			Size____________________
(01)	Even	________ (yes/no/NA)	(a) <1/4 inch (b) =>1/4 inch
(02)	Uneven	________ (yes/no/NA)	(a) <1/4 inch (b) =>1/4 inch
(03)	Vertical	________ (yes/no/NA)	(a) <1/4 inch (b) =>1/4 inch
(04)	Horizontal	________ (yes/no/NA)	(a) <1/4 inch (b) =>1/4 inch

C. Air Exchange System/ Appliances

19. Does the home have an exhaust fan which blows air out of the home?

(01) Yes _______ Location: _______ Number: _______

Yes _______ Location: _______ Number: _______

Yes _______ Location: _______ Number: _______

(02) No _______

(03) Other ____

20. If there is an exhaust fan, is it used?

(01) Yes ________

(02) No ________

(03) Other ________

21. The degree to which outside air is coming into the living space?
    *Approximate diameter (inches/feet)

Estimated diameter scale: (a) Small 6 in. (b) Medium 12 in. (c) Large 18 in.

Functional? Yes	No	Description	No. of Fans	Estimated Diameter	Self-Vented (Y/N/U)
☐	☐	Whole house exhaust	________	________	________
☐	☐	Fan over oven	________	________	________
☐	☐	Bathroom ventilation	________	________	________
☐	☐	Exterior air conditioning	________	________	________
☐	☐	Other	________	________	________
☐	☐	Other	________	________	________

22. Is there a woodstove in the home?
    (01) Yes ________
    (02) No ________
    (03) Other ________________________________

23. Is there a fireplace in the home?
(01) Yes ________
(02) No ________
(03) Other ____

24. If there is a fireplace in the home is it closed when not in use?
(01) Yes ________
(02) No ________
(03) Unknown ________

D. Sources of Indoor Radon

25. Is drinking water stored in the home?

		Quantity
(01)	Yes	________________
(02)	No	________________
(03)	Other	____

26. Source of water
(01) Water well ________
(02) Public source ________
(03) Unknown ________

E. Roof

27. Ceiling surface conditions

		Approximate Size (length, width)	Open/Closed?
(01)	Openings	_______________	___________
(02)	Cracks	_______________	___________
(03)	Skylight	_______________	___________
(04)	Other	_______________	___________
